# Associated risk factors and management of chronic diabetic foot ulcers exceeding 6 months’ duration

**DOI:** 10.3402/dfa.v3i0.18980

**Published:** 2012-10-30

**Authors:** Hassan Gubara Musa, Mohamed ElMakki Ahmed

**Affiliations:** 1 Jabir AbuEliz Diabetic Center, Khartoum, Sudan; 2 Department of Surgery, Faculty of Medicine, University of Khartoum, Sudan

**Keywords:** diabetic foot, ulcer, amputation, neuropathy, ischemia

## Abstract

**Background:**

The management of chronic diabetic foot ulcers (DFU) poses a great challenge to the treating physician and surgeon. The aim of this study was to identify the risk factors, clinical presentation, and outcomes associated with chronic DFU>6 months’ duration.

**Methods:**

This prospective study was performed in Jabir Abu Eliz Diabetic Centre (JADC), Khartoum, Sudan. A total of 108 patients who had DFU for >6 months were included. Recorded data included patient's demographics, DFU presentation, associated comorbidities, and outcomes. DFU description included size, depth, protective sensation, perfusion, and presence of infection. Comorbidities assessed included eye impairment, renal and heart disease. All patients received necessary local wound care with sharp debridement of any concomitant necrotic and infected tissues and off-loading with appropriate shoe gear and therapeutic devices.

**Results:**

The mean age of the studied patients was 56+SD 9 years with a male to female ratio of 3:3.3. The mean duration of DFU was 18±SD 17 months (ranging from 6 to 84 months). Ulcer healing was significantly associated with off-loading, mainly the use of total contact cast (TCC) (*p*=0.013). Non-healing ulcerations were significantly associated with longer duration of the chronic DFU>12 months (*p*=0.002), smoking (*p*=0.000), poor glycemic control as evidenced by an elevated HbA1c (>7%), large size (mean SD 8+4 cm), increased depth (*p*<0.001), presence of skin callus (*p*<0.000), impaired limb perfusion (*p*=0.001), impaired protective sensation as measured by 10 g monofilament (*p*=0.002), neuroischemia (*p*=0.002), and Charcot neuroarthropathy (*p*=0.017).

**Discussion:**

Risk factors associated with chronic DFU of>6 months’ duration included the presentation of an ulcer with increased size and depth, with associated skin callus and neuroischemia, in a diabetic patient with a history of smoking and increased HbA1c >7%. Off-loading mainly with the use of TCC is an effective method of managing long-standing DFU.

Diabetic foot ulcers (DFU) and concomitant infections are one of the most frequent complications in patients with diabetes mellitus (1, 2). DFU affect approximately 12–25% of persons with diabetes mellitus throughout their lives (3). Lower limb disease is the most common source of complications and hospitalization in the diabetic population (4). Diabetics (7–10%) develop chronic foot ulcers, a severe and expensive complication with life and/or limb threatening conditions (5). Chronic DFU are one of the most common indications for hospitalization in diabetics, and almost 50% of all non-traumatic amputations are performed on diabetic patients (6). It is now understood that the majority of diabetic lower extremity amputations are preceded by foot ulcers (1, 7).

Chronic DFU do not follow the well-described sequence of wound healing. Recent research has shown that true chronic wounds are biochemically different from acute wounds differing in their expression of growth factors, matrix metalloproteases, and various proteins. The most frequent chronic wounds are DFU, that is, ulcers due to peripheral arterial occlusive disease and/or venous disease (8, 9).

A chronic DFU is defined as a wound failing to heal after 4 weeks (9) and this definition was adopted by the American Diabetes Association (10). It has been reported that a decrease in wound size by at least 0.7 mm per week is 80% sensitive and specific for ultimate wound closure, the opposite being a marker of chronicity (11). Alternatively, a less than 10% decrease in wound surface per month may empirically be a predictor of poor healing, although reliability and predictive values are missing (8–11).

The aim of this study was to identify the risk factors, clinical presentation, and outcomes associated with chronic DFU of >6 months’ duration.

## Patients and methods

This prospective study included 108 patients who had chronic DFU for more than 6 months and was conducted in the Jabir Abu Eliz Diabetic Centre (JADC) in Khartoum, Sudan, from June 2010 to June 2011. Recorded data included patient's demographics, DFU presentation, associated comorbidities and outcomes. Exclusion criteria included DFU <6 months’ duration or patients that were not interested in participating in the study. The study was approved by an ethical committee in JADC.

All patients underwent a complete lower extremity examination and classification of their DFU according to the following five categories: perfusion, size, depth, presence of infection, and protective sensation. The perfusion assessment included evaluation of the presence of pedal pulses and measurement of the ankle brachial index (ABI) using a hand-held Doppler device. Peripheral arterial disease (PAD) was considered to be present if the ABI was <0.9 and/or both dorsalis pedis and posterior tibial pulses were absent. Limb ischemia was graded as shown in Table 1.

**Table 1:** Grading of lower extremity perfusion utilized for this study

Grade 1	No ischemia
	Palpable DP and PT
	ABI 0.9–1.1.
Grade 2	Intermittent claudication
	ABI < 0.9 but with a systolic ankle pressure >50 mm Hg.
Grade 3	Critical limb ischemia
	Systolic ankle pressure <50 mm Hg or toe pressure < 20 mm Hg.

DP = dorsalis pedis, PT = posterior tibial, ABI = ankle brachial index.

The size of the ulcer was determined by multiplying the largest by the second largest ulcer diameter and dividing it into three categories: <2 cm^2^, 2–5 cm^2^, and >5 cm^2^. The depth was described as either deep or superficial, if a full-thickness DFU was extending through the subcutaneous tissues or not. The grading system for the DFU depth is shown in Table 2. Peripheral neuropathy was defined as loss of protective sensation of a 10-g monofilament tested on the plantar aspect of the hallux, metatarsophalangeal joints 1 and 5, or loss of vibration sensation by using a 128 Hz tuning fork on the dorsum of the hallux.

**Table 2:** Grading for ulcer depth utilized for this study

Grade 1	Superficial ulcers not penetrating any structure below the dermis
Grade 2	Deep ulcers penetrating to the level of subcutaneous tissue, fascia, muscle, and tendon
Grade 3	Deep ulcers penetrating to the level of bone and/or joint

The site of the ulcer was divided into plantar (toes, forefoot, midfoot, and hindfoot) and non-plantar (dorsal, interdigital part of the toes and dorsal-lateral aspect of the foot and hindfoot). The ulcer duration was divided into three categories: 6–12 months, 12–24 months, and >24 months. Patient's comorbidities included the presence of severe visual impairment (i.e. cataract, retinopathy), presence of renal disease (hemodialysis, peritoneal dialysis, renal transplant), and ischemic heart disease.

All patients received local wound care, sharp debridement, antibiotics, and off-loading techniques as appropriate. The ulcer management included adequate drainage by sharp debridement of all necrotic and infected tissues or any abscess. The wound was left open for further assessment and treatment without any evidence of undermining or tracking. Deep infected tissues or bone fragments were sent for bacteriological cultures, and empiric intravenous antibiotic therapy was initiated by a third-generation cephalosporin that was changed, if necessary, according to the culture results and sensitivities. Proper glycemic control was maintained throughout the patient's management using short-acting insulin. The treatment protocol was consistent throughout the study period.

Data were collected using a pre-designed data collection sheet after getting informed consent from all patients. Data analysis was carried out using the statistical package for social sciences (SPSS v19) to compare healed versus non-healed DFU. Differences were tested by Chi-squared test and statistical significance was defined as 0.05.

## Results

Chronic DFU>6 months (*n*=108) accounted for 1.5% of all patients presented to JADC during the study period. A total of 65 patients (60%) had their ulcer healed and 42 were still on treatment during their 1-year follow-up (39.2%). One mortality incidence was reported from myocardial infarction. The mean age of all studied groups was 56+9 years and male to female ratio was 3:3.3. The mean ulcer duration was 18±SD 17 months with a range from 6 to 84 months.

Table 3 shows the general factors affecting DFU healing. Neither age (*p*=0.053) nor gender (*p*=0.871) ratio had an effect in DFU healing. A long-standing DFU with a mean duration of 28+9 months in a smoker with poor glycemic control (HbA1c> 7%) was unlikely to heal. In addition, the higher the grading system for perfusion, the worse the outcome of healing. A total of 53% and 75% of patients with grade 2 and 3 limb perfusion, respectively, did not heal (*p*<0.001). Fifty nine percent of ischemic ulcers did not heal (*p*=0.002). Neuropathic ulcers as diagnosed by impaired 10 g monofilament nylon were more likely to heal than neuroischaemic ulcers (*p*=0.002). Sixty five per cent of smokers did not have ulcer healing versus 35% of non-smokers (*p*=0.000). Ulcers with a mean duration of 28 months were unlikely to heal when compared with those with a mean duration of 18 months (*p*=0.002). However, the following factors had no effect on ulcer healing: alcohol consumption (*p*=0.189), type of diabetes (*p*=0.357), and type of anti-diabetic therapy (*p*=0.226). Associated comorbidities also did not have an effect on ulcer healing: hypertension (*p*=0.169), ischaemic heart disease (*p*=0.537), renal disease (*p*=0.167), and eye impairment (*p*=0.874).

**Table 3:** General factors affecting healing in chronic DFU >6 months

	Healed No. (%)	Not healed No. (%)	*p*
Mean age ± SD (years)	54.8±10.4	58.5±8.3	0.053
Male: female ratio	3	3.3	0.871
Mean duration of ulcer ± SD (months)	18.5±12.4	28.9±21.2	0.002*
Smoking	13 (35)	24 (65)	0.000*
HbA1c			
< 7%	33 (86)	5 (14)	0.000*
>7%	32 (46)	38 (54)	0.000*
Limb perfusion			
Grade 1	37 (80)	9 (20)	0.001*
Grade 2	27 (47)	31 (53)	0.001*
Grade 3	1 (25)	3 (75)	0.001*
Type of ulcer			
Neuropathic	30 (79)	10 (21)	0.002*
Neuroschemic	28 (41)	40 (59)	0.002*
Alcohol consumption	10 (76)	3 (24)	0.189
Duration of DM>20years	29 (58)	21 (42)	0.445
Type of DM			
Type 1	10 (71)	4 (29)	0 .357
Type 2	55 (58)	39 (42)	0.357
Glycemic control			
Insulin	60 (64)	36 (36)	0.226
OHD	3 (33)	6 (67)	0.226
Diet	1 (33)	2 (67)	0.226
Associated comorbidities			
Hypertension	18 (72)	7 (28)	0.169
IHD	3 (75)	1 (25)	0.537
Renal disease	2 (33)	4 (67)	0.167
Eye impairment	42 (61)	27 (39)	0.874

* *p* <0.05.

HbA1C = Glycated hemoglobin A1C, DM = diabetes mellitus, OHD = oral hypoglycemic drugs, IHD = ischemic heart disease

Table 4 shows the local factors affecting DFU healing. The mean size of the ulcer (±SD) was found to be significantly greater in those patients who did not heal (8±4 cm). Eight out of ten patients with an ulcer that penetrated the level of bone and/or joint (Grade 3) did not heal (*p*=0.001). Bacteriological examination revealed *Staphylococcus aureus* as the predominant isolant during the course of treatment in both groups without a significant effect on wound healing. Fourteen patients had a concomitant Charcot neuroarthropathy (CN), of which 10 did not heal (*p*=0.017) while all other foot deformities did not affect healing: clawtoes (*p*=0.853), pes cavus (*p*=0.653), crowded toes (*p*=0.809), hallux valgus (*p*=0.321), hammertoe (*p*=0.495), and other deformities (*p*=0.734). Twenty seven out of 35 patients using TCC had their ulcers healed (*p*=0.013). Forty eight patients did wear their therapeutic shoes, of whom 75% had their ulcers healed (*n*=36) (*p*=0.017). Fourteen out of 17 patients using a wheelchair had their ulcers healed (*p*=0.042). Diabetic patients using crutches regularly (*n*=24) had healing versus 9 patients who were not using them regularly (*p*=0.077). The presence of a skin callus was more frequent in those who did not heal compared with those who healed (*p*=0). Previous toe amputation did not affect the healing of chronic foot ulcers (0.692).

**Table 4:** Local factors affecting healing in chronic DFU >6 months’ duration

	Healed No. (%)	Not healed No. (%)	*p*
Ulcer depth			
Grade 1	20 (87)	3 (13)	0.001*
Grade 2	43 (57)	32 (43)	0.001*
Grade 3	2 (20)	8 (80)	0.001*
Staphylococcus aureus	19 (53)	17(47)	0.516
Skin callus	30 (42)	41 (58)	0.000*
Previous toe amputation	28 (61)	18 (39)	0.692
Foot deformity			
Charcot joint	4 (29)	10 (71)	0.017*
Clawtoe	13 (62)	8 (38)	0.853
Pes cavus	27 (63)	16 (37)	0.653
Crowded (overlapping) toes	18 (62)	11 (38)	0.809
Hallux valgus	14 (70)	6 (30)	0.321
Hammertoe	13 (54)	11 (46)	0.495
Other deformity	17 (63)	10 (37)	0.734
Off-loading			
Total contact cast	27 (77)	8 (23)	0.013*
Therapeutic shoes	36 (75)	12 (25)	0.017*
Wheelchair	14 (82)	3 (18)	0.042*
Crutches	24 (73)	9 (27)	0.077
Other	0	1	0.578

* *p*<0.05.

## Discussion

Treatment of DFU is complicated and healing may take several months or sometimes years (5), and our literature review revealed no reporting on this specific category of chronic DFU of >6 months’ duration. In our study, neither the patient's age, gender nor the type of diabetes had any effect in DFU healing. Ince et al. (11) reported similar findings where age, gender, and type of diabetes in addition to infection and peripheral neuropathy had no effect on healing of DFU of short duration (mean of 29 days) in the United Kingdom. Other publishers reported similar findings (12) in addition to the duration of diabetes and ulcer site, where both had no effect on ulcer healing (13).

The most significant general characteristics of a patient with chronic non-healing DFU in our study were ischemia, peripheral neuropathy, smoking, and elevated HbA1c. Peripheral vascular disease is considered to be a limiting factor for healing (14). However, this finding is not consistent in all studies. Parisi et al. reported no association between healing of a diabetic ulcer and peripheral vascular disease (15). This could be due to the fact that life expectancy of diabetics is lower in developing countries compared to the Western world. In the absence of vascular intervention in our center, peripheral vascular disease was the most significant indication for major lower extremity amputation. Similar data have been reported from other developing countries due to a lack of revascularization services (16).

The association between severe peripheral neuropathy and non-healing diabetic ulcers was reported from developing countries (15, 17). In our study, the combination of peripheral neuropathy and ischemia was the commonest predictor of non-healing DFU. Loss of sensation and PAD were associated with a poorer outcome in these patients, suggesting that loss of protective sensation is not only a key factor in the development of an ulcer but also affects its outcome. This may be related to the preserved mechanism of off-loading the ulcer in individuals with intact protective sensation. However, neuropathy may also have direct effects on wound healing with some studies suggesting that denervation may contribute to impaired wound healing in diabetes (18, 19). On the contrary, a large retrospective database analysis on individuals with neuropathic ulcers reported higher healing rates. These may be related to an increased awareness of the importance of adequate off-loading, as a result of publication of international guidelines and reports on casting techniques (20, 21). In the absence of health education and emphasis on diabetic foot care as being practiced in developing countries, peripheral neuropathy will continue to be a major component in delayed wound healing.

Smoking has been related to slow wound healing (22) and to insulin resistance (23), especially in those smoking more than 10 packs/year (24). A significant correlation was also noted between a history of smoking and increased HgbA1c levels (25). In our study, smoking was significantly common among the non-healing group of patients. The number of patients with medical comorbidities, for example, retinopathy, nephropathy, and cardiac disease was small and no significant association with outcome was observed. End stage renal disease and hemodialysis pose a high risk for foot ulceration and amputation; hence, those ulcers may not last long enough to attain chronicity (26, 27).

The local ulcer characteristics, that is, depth and presence of a skin callus had a significant effect on delayed wound healing. The callus in the diabetic foot ulcer has shown pathology similar to human papillomavirus but without its presence (28), and serial surgical debridements may increase the wound healing rate (29, 30). Parisi et al. also reported delayed healing in deeper ulcers of long duration (15). The deeper the ulcer is penetrating through the dermis, the more the tissue damage, necrosis, and osteomyelitis. Rooh et al. reported that those with higher grades of sepsis needed amputation (31). In addition, Imran et al. (32) stated that the frequency of minor and major amputations increases with the higher grades of diabetic foot ulceration. In our study, we reported the incidence of *Staphylococcus aureus* isolation during the course of treatment with and without manifesting the classical clinical systemic symptoms with a similar experience reported by other authors (33, 34). The absence of other isolates like anaerobes reflects the difficulties encountered in sampling and isolation by the traditional methods (35).

Total contact casting is commonly recognized as a great off-loading modality in healing DFU. Off-loading is the most important healing factor for chronic diabetic wounds (36) and there is evidence-based data to support its use (37, 38). A clinical trial by Wu et al. (39) found that the TCC resulted in higher healing rates and a shorter time to healing than a removable cast walker, with a similar capacity for off-loading wounds. However, the most important attribute of the TCC may be its ability to maintain patient compliance. The patient has little choice other than to adhere to the regimen prescribed by the clinician, because the device is not easily removable (39). The TCC helps to reduce or control edema and potentially protect the foot from infection (40). Seventy five percent of our patients using TCC for off-loading had their ulcer healed in less than 1 year while 25% did so in more than 1 year. Those using the TCC for more than 6 weeks (83%) had their ulcer healed.

In our study, 29% of all patients complied with therapeutic shoes and 75% of them had their ulcers healed (*p*=0.01). Despite its beneficial effects, compliance with preventative footwear is rather poor. A study looking into prescribed footwear use in the United Kingdom found that only 22% of their subjects wore the prescribed footwear all day (41). Other authors reported no effect with the use of conventional or standard footwear (38). Regular adherence to prescribed therapeutic shoes reduced the formation of skin callus around the ulcer. The presence of skin callus in the DFU was considered as evidence of high vertical load that could be prevented by off-loading; however, other factors might have played an important role. In addition, *n*=14 (13%) of our patients had CN, of which two-thirds had non-healing ulcerations. The rocker-bottom deformity associated with CN is prone to increased pressure and ulceration and is more resistant to healing. Wound closure may be difficult to achieve with local wound care and off-loading techniques, if the predisposing deformity that caused the ulceration is not addressed (42).

Edmond et al. (43) reported that heel ulcers tend to be ischemic, with relative paucity of vessels and hence take a longer time to heal. Heel ulcers may be considered to have the poorest prognosis among DFU attributed by fat pad atrophy in people with diabetic neuropathy, reducing the heel's cushioning ability (44). Bakheit et al. reported a favorable outcome in patients with superficial heel ulcers and adequate limb perfusion (45), and similarly in this study, heel ulcers were not associated with non-healing.

With regards to the overall outcomes, our study showed that 60% of DFU healed, 39.2% were still under treatment, and one death due to myocardial infarction was reported. Many studies found relatively comparable outcomes; however, the duration of those ulcers were not uniformly exceeding 6 months. A more recent study (46) showed that within a 1-year period of follow-up, 77% of patients healed, 12% were still under treatment, and 6% died. Jeffcoate et al. (47) reported healing rates of 66% and a German cohort study by Beckert et al. (48) found healing rates between 57% and 93%.

The costs of chronic ulcer care represent a major portion of the health care budget and continue to grow at exponential rates and this is an important issue in developing countries (49). Prevention in the form of health education, diabetic foot care, and footwear will be the most effective tools to avoid chronic DFU. Our study limitations included the lack of patient's recorded body mass index, type of wound dressing, and patient compliance. In addition, frequent complications such as sepsis were also not reported in our study. In conclusion, risk factors associated with long-standing DFU >6 months’ duration included a large and deep ulcer with a skin callus, an ulcer of longer duration, inadequate limb perfusion, impaired protective sensation with an elevated HbA1c, and history of smoking. Off-loading using TCC was an effective adjunctive method of managing chronic DFU.
